# The ‘Goldilocks Zone’: Getting the Measure of Manual Asymmetries

**DOI:** 10.1371/journal.pone.0128322

**Published:** 2015-05-29

**Authors:** Rachael K. Raw, Richard M. Wilkie, Alan White, Justin H. G. Williams, Mark Mon-Williams

**Affiliations:** 1 School of Psychology, University of Leeds, Leeds, United Kingdom; 2 Division of Applied Health Sciences, University of Aberdeen, Clinical Research Centre, Royal Cornhill Hospital, Aberdeen, United Kingdom; Goldsmiths, University of London, UNITED KINGDOM

## Abstract

Some studies have shown that manual asymmetries decrease in older age. These results have often been explained with reference to models of reduced hemispheric specialisation. An alternative explanation, however, is that hand differences are subtle, and capturing them requires tasks that yield optimal performance with both hands. Whereas the hemispheric specialisation account implies that reduced manual asymmetries should be reliably observed in older adults, the ‘measurement difficulty’ account suggests that manual asymmetries will be hard to detect unless a task has just the right level of difficulty—i.e. within the ‘Goldilocks Zone’, where it is not too easy or too hard, but just right. Experiment One tested this hypothesis and found that manual asymmetries were only detected when participants performed in this zone; specifically, performance on a tracing task was only superior in the preferred hand when task constraints were high (i.e. fast speed tracing). Experiment Two used three different tasks to examine age differences in manual asymmetries; one task produced no asymmetries, whilst two tasks revealed asymmetries in both younger and older groups (with poorer overall performance in the old group across all tasks). Experiment Three revealed task-dependent asymmetries in both age groups, but highlighted further detection difficulties linked with the metric of performance and compensatory strategies used by participants. Results are discussed with reference to structural learning theory, whereby we suggest that the processes of inter-manual transfer lead to relatively small performance differences between the hands (despite a strong phenomenological sense of performance disparities).

## Introduction

Most humans have a strong phenomenological sense that one of their hands is superior to the other hand when carrying out most motor tasks (e.g. writing, throwing a ball). This preferential use of the right or left hand develops during childhood and is presumed to be maintained throughout life [1]. Many studies of young adults have reported superior performance of the preferred hand across a range of motor activities [2–4]. It seems reasonable, therefore, to assume that the measurement of manual dexterity will reveal differences between the two hands of most individuals. But is this a safe assumption?

Our current understanding of how manual asymmetries change with age is limited, and given the overwhelming evidence for an age-related decline in general motor function [5–11], it seems possible that old age might lead to differential changes in the capabilities of the preferred and non-preferred hands. For example, older people may become increasingly dependent on using the preferred hand due to many years of practice. Alternatively, discrepancies between the hands might diminish with age as a consequence of neurological changes; specifically that reduced hemispheric lateralisation and an increased bilateral pattern of cortical activation in the motor areas of the aging brain leads to reduced manual asymmetries for older adults performing movement tasks [12–15]. This hemispheric lateralisation account has gained considerable attention over recent years; however evidence varies greatly, both with regards to the nature of age differences detected at a neurological level, and the degree of manual asymmetries identified in behavioural experiments. For example, neurophysiological studies of older adults have found reduced hemispheric asymmetry in the motor cortex, and the recruitment of additional brain regions during finger-tapping, button-pressing and hand-grip tasks [16–18], yet this does not seem to extend to implicit motor sequence learning or cued simple movements [19, 20]. There is also a conflict in the behavioural evidence. In some studies, older adults display reduced asymmetries in reaching, visuomotor adaptation and fine motor control [15, 20–22], but other studies have reported no age differences in asymmetries on different motor tasks [23–26], or even an increase in older adult asymmetries compared to the young [24, 26, 27–29].

The empirical picture in older adults is undoubtedly confused—and similar conflict can be seen in a number of studies that have made claims based on the observation of reduced or absent motor asymmetries in special populations. Such claims have a long history; for example Orton [30] argued that reduced manual asymmetries indicate an underlying confusion of cerebral dominance in children with developmental disabilities—an argument that remains in the developmental literature to this day [31]. An improved understanding of the variety of results in studies of manual asymmetries therefore has practical and clinical implications, in particular for informing whether we are safe in assuming there will be superior performance by the preferred hand across a range of tasks.

Whilst preferred hand advantage is phenomenologically strong (i.e. it can feel more awkward to perform certain actions with the non-preferred hand), we would suggest that manual asymmetries are likely to be fairly subtle. The lifelong effects of Structural Learning (SL: a generalised learning effect whereby mastering a skill with one hand will allow a high level of performance in the other hand [32]) should lead to minimal asymmetries. According to SL theory, the human nervous system acquires general rules that can be readily applied when controlling actions with similar dynamics [32]. A canonical example is learning to ride a bicycle—once the general rules about the control dynamics of the action are learned, it is very quick to learn how to ride a new bicycle with different characteristics [33]. Numerous studies have provided evidence for the existence of SL in the motor system. Yousif and Diedrichsen [34] found that the motor system uses previously learned temporal structures when moving in novel force fields, and exploits knowledge of these structures to improve adaptation to new perturbations. Furthermore, Braun et al. [33] demonstrated that adaptation to horizontal or vertical visuomotor rotations is facilitated by previous exposure to the structure of such perturbations. SL is also evident in fine motor control tasks, such as tracing. Johnson, Culmer, Burke et al. [35] compared performance when participants traced shapes leftwards versus rightwards. Tracing performance was found to be better when moving in the conventional Western handwriting direction (i.e. rightwards) for both the preferred and non-preferred hands, and in both the right-handed and left-handed participants. These results are a prime example of SL being evident in everyday motor tasks.

Structural Learning (SL) predicts that learning a particular skill with one hand will allow the nervous system to learn the dynamics that underpin that particular skill. This suggests that there can be extensive inter-manual transfer after a skill is mastered with one hand, as the other hand will benefit from the learned structure of the task. The findings of Johnson et al. [35] show that the dynamics of writing with the preferred hand influences the behaviour of the non-preferred hand. Hence, it follows that performance differences between the hands will be relatively small—even when a task has been practised only with the preferred hand. Nevertheless, it should not be the case that the non-preferred hand would be capable of equivalent performance to the preferred hand. Wolpert et al. [32] argue that there are at least three levels of representation that are relevant when learning a task. Namely, these are the ‘task structure’, the ‘task parameters’ and the ‘task-relevant state information’. For example, the structure of holding a pen to produce handwriting can be learned from the preferred hand, allowing a reasonably high level of performance to be observed in the non-preferred hand. But the precise parameters of the task structure (i.e. the biomechanics of the arm when holding the writing implement) will also need to be learned to control the non-preferred hand. If task parameter estimates are based on the biomechanics of the preferred hand then this could actually impede performance and might also be expected to hinder estimation of task-relevant state information. Structural Learning (SL) can therefore explain why participants are able to write with their non-preferred hand to a reasonable level of performance, rapidly improve performance after intensive training, but show reduced levels of performance relative to the preferred hand without the requisite training.

The evidence supporting SL theory leads to the hypothesis that there will be some degree of difficulty when it comes to detecting manual asymmetries in experimental tasks (i.e. because absolute differences between the hands will be relatively small). It also follows that asymmetries will only become apparent when participants are pushed to the limits of their performance capability, a threshold which will inevitably vary both across individuals and between groups. If the task is too difficult then it will be hard to differentiate the preferred and non-preferred hands (i.e. both hands will show a floor effect). If the task is not difficult enough, both hands will perform at ceiling levels. Hence the detection of asymmetries requires tasks that are in the ‘Goldilocks Zone‘(i.e. not too easy or too difficult, but just right), whereby a sufficient level of complexity is present to demonstrate the superior performance of the preferred hand, without being so difficult that neither hand can perform well. Essentially, the lifelong processes of SL provide a plausible explanation for the variable outcomes identified in behavioural studies of manual asymmetries, given the assumption that not all tasks would have placed participants within the ‘Goldilocks Zone’.

In order to empirically test the concept of the ‘Goldilocks Zone’, and its implications for the measurement of manual asymmetries, our first experiment used a tracing task where the speed of participants’ tracing was constrained under two conditions. The prediction was made that manual asymmetries would be seen when a fast tracing speed constraint was used, but would not be seen with a slower speed constraint (because this made the task too easy). We were also interested in testing this hypothesis within a young adult population—few studies have examined the issue of measurement difficulties in young or middle-aged adults (i.e. from 18–40 years), and there are mixed findings across the ageing and developmental literature with regards to the nature of manual asymmetries.

Experiment Two extended our examination of the ‘measurement difficulty’ hypothesis to see if it also captured the behaviour of older adults. A major limitation of previous research into manual asymmetries in older adults is that most studies used outcome measures that combined performance speed and accuracy (resulting in a single overall score for performance), or only tested movement speed [17, 23–25, 28, 27]. Relying solely on one metric could mean that these experiments failed to identify asymmetries that were manifest in other aspects of performance. In Experiment Two, therefore, we used a battery of tasks that varied in content and complexity (tracking, aiming and tracing movement tasks) and recorded a number of outcome measures to capture performance differences in both younger and older adults.

A further implication of metric choice in the detection of manual asymmetries relates to the tendency of humans to apply strategies (cognitive and/or motor) to minimise task constraints and/or compensate for motor decline (e.g. age-related). This has consequences for detecting manual asymmetries given that participants might adopt a strategic approach that moves them away from the ‘Goldilocks Zone’. This is particularly problematic when comparing age groups, as different groups may have diverse strategic approaches based on their underlying abilities. For example, older adults have been found to make both temporal and spatial adjustments to their movements in order to meet task demands [8, 22, 36, 37]. The final experiment therefore explored the extent to which strategic speed-accuracy trade-offs caused difficulties in the detection of manual asymmetries.

## Experiment One

### Method

#### Participants

Thirty healthy young adults with no history of ophthalmological or neurological problems (self-reported) formed an opportunistic sample. The group comprised 19 males and 11 females aged between 18 and 39 years-old (mean = 29.4, *SD* = 7.77), recruited from the local Leeds community. All participants were right-handed as indexed by the Edinburgh Handedness Inventory (EHI) [38] with a mean score of 94.7 (*SD* = 9.05) out of the maximum score of 100. All participants gave their written informed consent, and the University of Leeds Ethics and Research committee approved this study, which was performed in accordance with the ethical standards laid down in the 1964 Declaration of Helsinki (NB. this statement applies also for Experiments Two and Three).

#### Materials and Procedure

**Fig 1:**
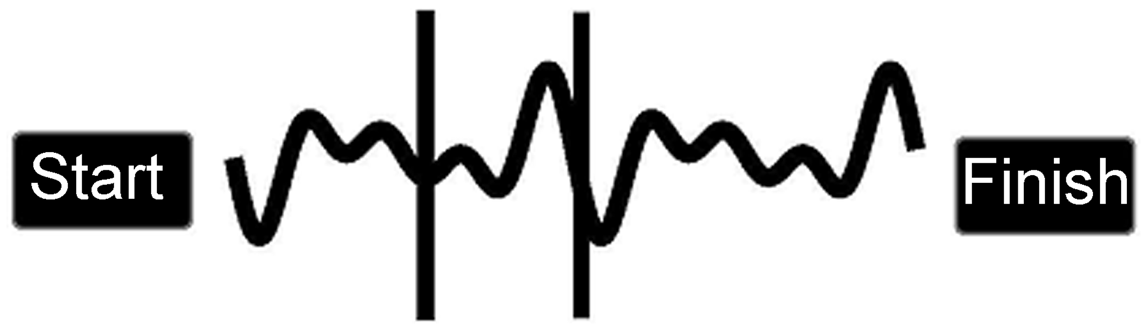
KineLab Tracing Task (Experiment One). Screen shot taken from the KineLab tracing task in Experiment One (NB. not to scale). The ‘narrow’ path thickness condition is illustrated in this example.

Two dynamic tracing tasks were created using ‘KineLab’ [2], a sophisticated digitised kinematic assessment tool that can be used to design visual-spatial tasks and record the kinematics of the hand controlling the input device. Participants used a handheld stylus, which was the same shape as a ballpoint pen (stylus length = 150mm; nib length = 1mm) to complete a path tracing task that was delivered on a tablet PC (screen 260 x 163mm; 1,440 x 900 pixels; 32 bit colour; 60 Hz refresh rate; see Fig 1). Each path was the same shape (height top to bottom = 184.3mm; width left to right = 19.8mm), but varied in thickness (2mm, 4mm, 6mm), in order to manipulate spatial constraints. To examine behaviour under different temporal constraints, the timing of the task was also precisely controlled. A constant speed was set by asking participants to trace within a moving window: two horizontal red bars (spaced 250mm apart) that gradually progressed along the path during trials (NB. choice of speed parameters based on previous work with a similar tracing task [22]). The two tracing tasks were hence:

- Fast speed tracing (23.64 mm/sec)
- Slow speed tracing (12.86mm/sec)

Each path thickness condition (i.e. narrow, medium and thick) was presented five times within each of the speed conditions (i.e. in the fast and slow versions) resulting in a total of 30 paths to trace. These conditions were presented in a different, random order for each participant, and in all conditions the path remained static and was fully visible throughout the trial. Participants were asked to complete the task once using their preferred (right) hand and once using their non-preferred (left) hand, which was counterbalanced so that every other participant began with their non-preferred hand. The instructions that appeared on the screen at the start of the task read “*follow the path from start to finish*, *remaining within the two red bars*. *You must not go outside of the path*”. The outcome measure of interest was Shape Index (SA) which indicates the extent to which the traces produced by participants matched the shape of the path. To determine Shape Error (SE), a 'point-set' registration technique was used to compare the path made by the participant (i.e. the 'input' path) with a given reference (i.e. the centre of the path stimulus). Point-sets were generated for the input and reference paths by discarding temporal information and re-sampling the X and Y coordinates at a spatial resolution of 11mm using linear interpolation. A robust point-registration method [39] that we have applied in previous studies [22, 36] was then used to determine the rigid transformation that best transformed the input path to match the reference paths, with SE scores being the mean distance between these points on these two paths. This measure is a particularly useful marker of accuracy, because it shows the degree to which participants remained within the path boundaries and how far participants deviated from the shape of the path stimulus. Importantly higher SE scores indicate greater deviation from the reference path, and hence reflect reduced accuracy.

#### Analysis

Mean SE performance scores in the three path thickness conditions on both the slow and fast versions of the task were calculated (i.e. SE for Fast and Slow speed tracing). A repeated measures ANOVA was applied in order to examine whether tracing accuracy varied between the preferred and non-preferred hands for different path thicknesses and/or speed conditions (Slow and Fast). Greenhouse-Geisser estimates of sphericity (ε) are reported where degrees of freedom have been adjusted.

### Results

**Fig 2:**
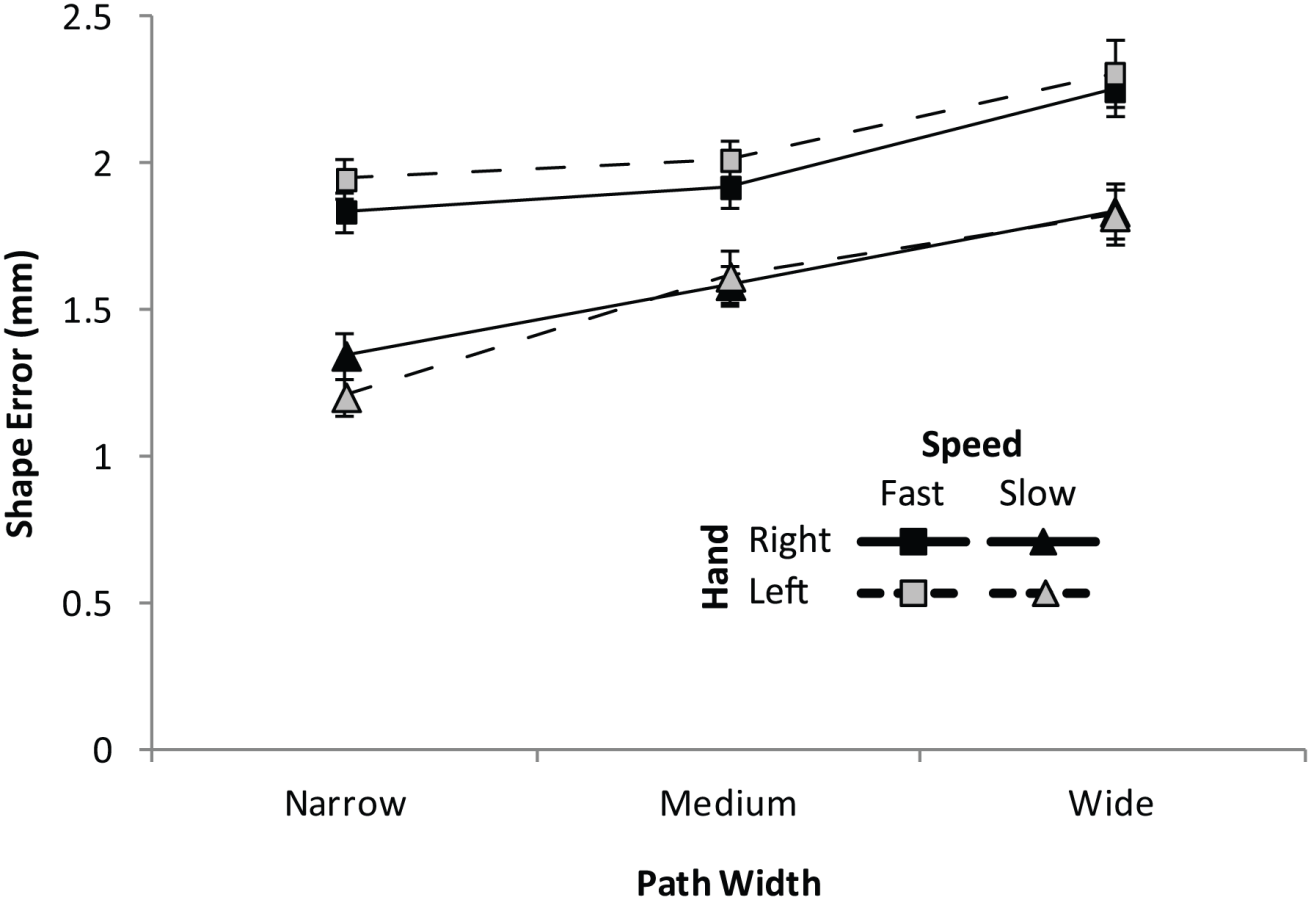
Shape Error (SE) in KineLab Tracing Task (Experiment One). Shape Error (SE; mm) when tracing paths of variable thickness in Experiment One with either the preferred (right) hand (black symbols and solid lines) or non-preferred (left) hand (gray symbols and dotted lines); either when moving at fast speeds (square symbols) or slow speeds (triangular symbols). Higher scores denote reduced accuracy. The bars represent the standard error of the mean (SEM).

The ANOVA for SE revealed a main effect of speed (*F* (1, 27) = 455.08, *p* < .001, *η* ^*2*^_*p*_ = .94), path thickness (*F* (2, 54) = 97.98, *p* < .001, *η* ^*2*^_*p*_ = .78, *ε* = .65) and a speed x width interaction *(F* (2, 54) = 17.41, *p* < .001, *η* ^*2*^_*p*_ = .39). While there was no significant main effect of hand (*F* (1, 27) = .64, *p* = .43, *η* ^*2*^_*p*_ = .02), crucially there was a hand x speed interaction (*F* (1, 27) = 11.23, *p* < .001, *η* ^*2*^_*p*_ = .29) whereby hand differences in SE were apparent at fast, but not slow, speeds. This finding is further supported by the absence of a hand x path thickness interaction (*F* (2, 54) = .88, *p* = .42, *η* ^*2*^_*p*_ = .03, ε = .807) and a lack of a 3-way interaction (*F* (2, 54) = 1.43, *p* = .25, *η* ^*2*^_*p*_ = .05, *ε* = .765). When inspecting the direction of the hand x speed effect it is evident that the preferred (right) hand improved in accuracy as compared to the non-preferred (left) hand when movements were fast, particularly during narrow and medium width conditions (narrow condition; *t* (28) = 2.51, *p* = .009; medium condition: *t*(29) = 1.78, *p* = .043). In contrast, at slow speeds, there is no indication of an effect in this direction, with the preferred hand being significantly *less accurate* for narrow paths (*t* (29) = 3.37, *p* = .001); see Fig 2).

### Discussion

The findings of Experiment One demonstrate that it is possible to elicit hand asymmetries when using a task of ‘sufficient’ complexity. Structural Learning (SL) theory predicts that the non-preferred hand should be capable of carrying out a motor task that has similar control dynamics to those acquired previously when using the preferred hand. The simple task of tracing a line at a very slow pace meant that participants were able to match the accuracy of their non-preferred hand even under increasing levels of spatial constraint. When speed was increased to a faster pace, however, differences between the hands were apparent, with the preferred hand showing superior spatial accuracy. In this experiment, the ‘Goldilocks Zone’ was only reached when temporal constraints were high. A task with identical spatial conditions fell outside of the ‘Goldilocks Zone’ when speed was kept relatively slow. These findings demonstrate that even in young healthy adults (where preferred hand performance is expected to be superior), the underlying characteristics of the task (i.e. in this case, the spatial and temporal demands) can mediate whether manual asymmetries are detected.

## Experiment Two

The first experiment highlighted important difficulties that can arise when designing studies to measure differences in motor performance between the preferred and non-preferred hand—specifically the spatial and temporal characteristics of a motor task. In Experiment Two, we used a battery of motor tasks to establish whether similar task-specific effects would also influence the detection of age differences in manual asymmetries. Critically, the test battery allowed asymmetries to be tested under a number of different measurement conditions including tracking, aiming and tracing tasks, which each varied in degree of spatial and temporal constraint, and the outcome metric used to capture performance.

### Method

#### Participants

A new opportunistic group of eighty five healthy right-handed individuals (mean EHI = 97.72; *SD* = 7.47), with no previous history of ophthalmological or neurological problems (self-reported), was recruited (from the University of Leeds Chaplaincy). The ‘young’ group (33 females, 34 males) were aged between 18 and 40 years (mean = 23.59, *SD* = 4.68) and the ‘old’ group (10 females, 8 males) were aged between 60 to 83 years (mean = 70.89, *SD* = 4.95).

#### Materials and Procedure

**Fig 3:**
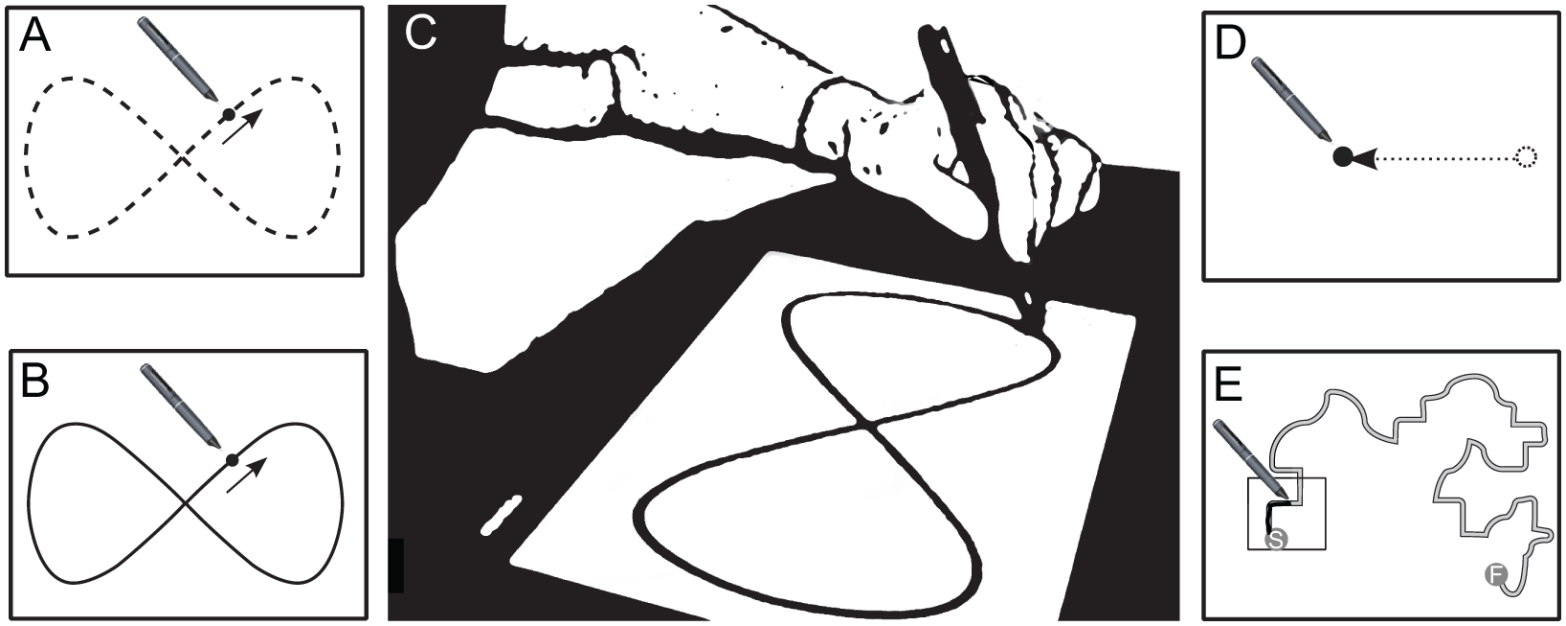
KineLab Motor Battery Tasks (Experiment Two). Screen shots taken from the KineLab motor task battery in Experiment Two (NB. not to scale), which included Manual Tracking without (A) and with (B) a spatial pattern, Aiming (D), and Tracing (E). (C) shows an older adult completing the Manual Tracking Task (with spatial pattern).

A battery of motor tasks was created with ‘KineLab’ [2], which participants completed using their preferred (right) hand and non-preferred (left) hand in a counterbalanced order. The three tasks were as follows;

- Manual Tracking: Participants were instructed to keep the stylus on a green dot that moved around the screen in a figure-of-eight pattern (dot diameter = 10mm). The speed of the dot progressed from a slow pace whereby it took 16sec to complete one figure-of-eight, to a medium (time = 8sec), and fast (time = 4sec) pace respectively. Each speed condition repeated three times before increasing to the next speed, resulting in a total of nine figure-of-eights to track (see Fig 3a). Immediately after these trials, participants followed the same instructions but with the spatial pattern visible throughout (a line drawn figure-of-eight shape, height = 110mm; width = 55mm; see Fig 3b). This task required participants to match the changing spatial location of the target. Root Mean Square Error (RMSE) was therefore chosen as the outcome variable, as it provides a single metric of performance accuracy. RMSE (mm) is the average distance of the stylus from the closest reference point in the centre of the figure-of-eight path: a higher RMSE value indicates reduced accuracy.
- Aiming: Participants were instructed to move the pen as quickly as possible from one green dot (diameter = 10mm) to another as each one appeared on the screen (distance between dots = 117mm). The appearance of the dots followed the shape of a pentagram which repeated 10 times (5 moves per repetition) (see Fig 3d). As this task required participants to move from one fixed position (of defined spatial accuracy) to another at a rapid pace, decreasing movement duration was the challenge of the task. Accordingly, Movement Time (MT), the time taken to move the stylus between two dots, was calculated across all of the aiming movements and the mean MT used as the measure of performance (i.e. where higher MT indicates reduced performance).
- Tracing: Participants were required to trace a complex path (height = 166mm; width = 132mm; thickness = 4mm) from start to finish whilst trying to remain within the section of the path highlighted by a translucent box. The box changed position in steps to progress around the path (a change every 5sec), in order to enforce a steady pace and constrain the MTs of participants. There were six trials which featured two versions of the path, the second version being a mirror-image of the first path, which appeared every other trial (see Fig 3e). Because MTs were controlled, Shape Error (SE) was used as a measure of performance accuracy. The SE metric was calculated by taking each traced path and calculating the error relative to a reference path that marked the exact centre. This was achieved using an automated ‘point-set registration’ technique that is described in Raw et al. [22]. Note that higher SE values indicate greater deviation from the reference path, and a reduced level of accuracy.

#### Analysis

Mixed model ANOVAs were used to examine the effects of age and hand on the mean scores for all outcome measures recorded in each task (RMSE, MT and SE). For the Aiming and Tracing tasks the mean scores across all trials were calculated, and separate ANOVAs applied. Further specifics on the analysis for the Manual Tracking data are detailed in the results section. For all analyses, Greenhouse-Geisser estimates of sphericity (ε) are reported where degrees of freedom have been adjusted.

### Results

#### Manual Tracking

**Fig 4:**
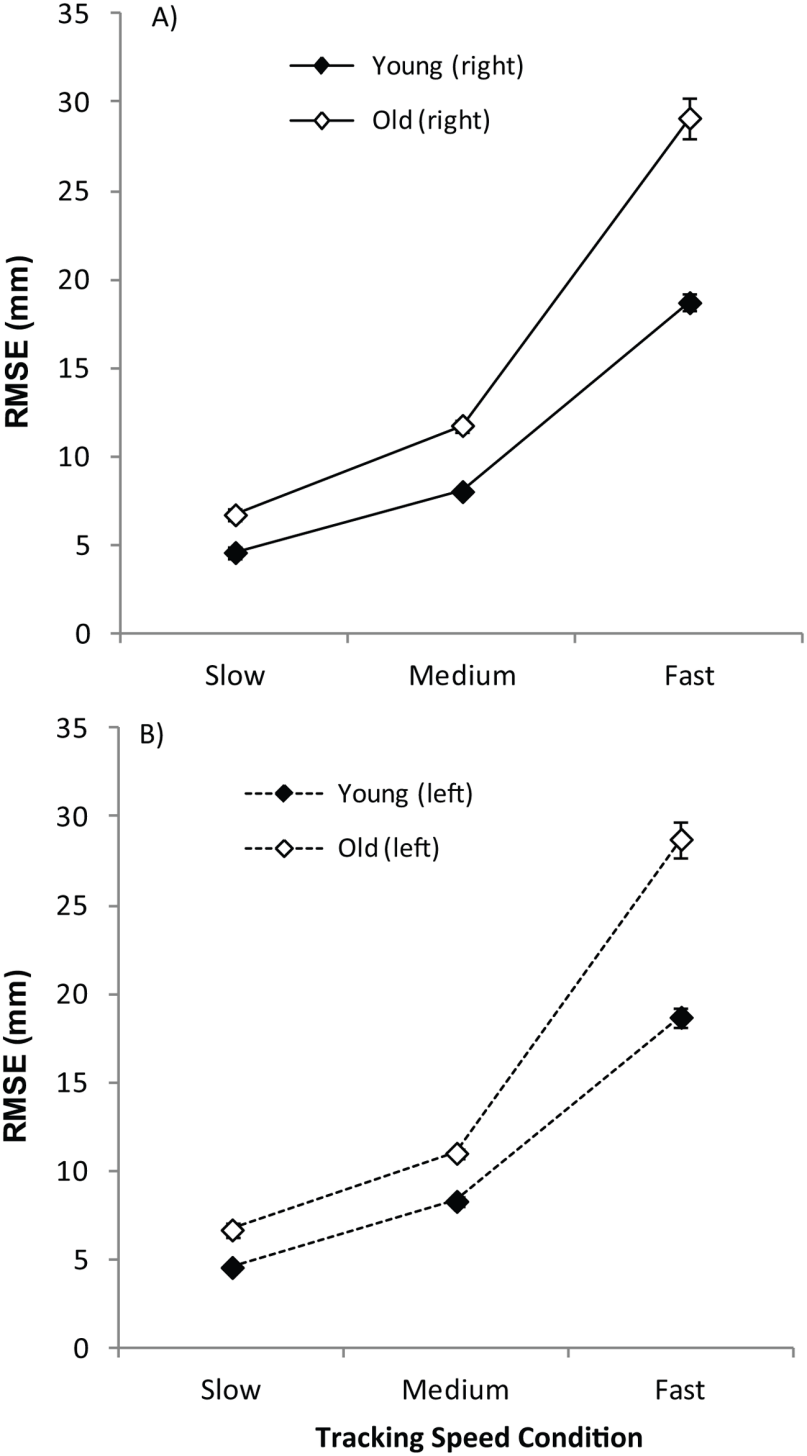
Root Mean Square Error in Manual Tracking Task (Experiment Two). Mean Root Mean Square Error (mm) in the Manual Tracking Tasks in Experiment Two, for the non-preferred left (dashed lines) and preferred right (solid lines) hand, in the old (open symbols) and young (filled symbols) groups. Larger RMSE values indicate reduced accuracy. The bars represent the standard error of the mean (SEM).

An initial analysis of Root Mean Square Error (RMSE) data from The Manual Tracking Tasks showed that while there the spatial pattern led to significant improvements in performance (*F*(1, 79) = 44.41, *p* <.001, *η* ^*2*^_*p*_ = .36) the direction of main effects for speed, hand and age were the same for both versions of the task—with and without the spatial pattern (no interaction between spatial pattern × hand: *F*(1, 79) = .87, *p* = .36; no interaction between spatial pattern × hand × age: *F*(1, 79) = .31, *p* = .58; and no interaction between spatial pattern × hand × speed: *F*(1, 78) = .93, *p* = .40). To simplify reporting of the findings, we therefore collapsed the data across the two Manual Tracking Tasks, and report the statistics from the combined versions. The results showed that tracking became less accurate as the speed of the dot increased (*F* (2, 166) = 1361.80, *p* < .001, *η* ^*2*^_*p*_ = .94, *ε* = .62). Older participants were less accurate than the young (*F* (1, 83) = 94.01, *p* < .001, *η* ^*2*^_*p*_ = .53), with a significant speed x age interaction highlighting a disproportionate effect of task difficulty on accuracy in the older group (*F* (2, 166) = 72.22, *p* < .001, *η* ^*2*^_*p*_ = .47, *ε* = .81). Thus, it can be seen in Fig 4 (which displays mean RMSE for the old and young in the slow, medium and fast speed conditions) that accuracy scores in the old group moved further away from the scores achieved by the young as the speed of the dot increased (i.e. the difference in mean RMSE between the old and young in slow condition = 2.14mm; medium = 3.22mm; fast = 10.16mm with increased effect size between young and old as speed increased (slow condition: *t* (83) = 4.97, *r* = .479; medium condition: *t* (83) = 7.89, *r* = .655; fast condition: *t* (83) = 9.87, *r* = .73). Crucially there was no significant main effect of hand, and there was no hand x age, speed x hand, or speed x hand x age interactions (all *p* > .05).

#### Aiming

The ANOVA for Movement Time (MT) in the Aiming Task established a between-participant effect of age whereby the old were slower than the young (*F* (1, 83) = 67.32, *p* < .001, *η* ^*2*^_*p*_ = .45). A main effect of hand also revealed manual asymmetries (*F* (1, 83) = 6.14, *p* = .015, *η* ^*2*^_*p*_ = .07), with participants producing faster aiming movements when the preferred hand was used. There were, however, no hand x age, speed x hand or speed x hand x age interactions (all *p* > .05).

### Tracing

The Tracing Task applied both spatial and temporal constraints on movement. The analysis for the Shape Error (SE) measure showed that the old were less accurate (*F* (1, 83) = 39.19, *p* < .001, *η* ^*2*^_*p*_ = .32), with higher SE scores than the young (mean SE for old = 1.19mm; mean SE for young = 0.89mm). Manual asymmetries were also identified (*F* (1, 83) = 23.46, *p* < .001, *η* ^*2*^_*p*_ = .22) whereby tracing was more accurate when the preferred hand was used. There were no significant interactions between hand x age, speed x hand or speed x hand x age (all *p* > 0.05).

#### Individual differences in manual asymmetries

**Fig 5:**
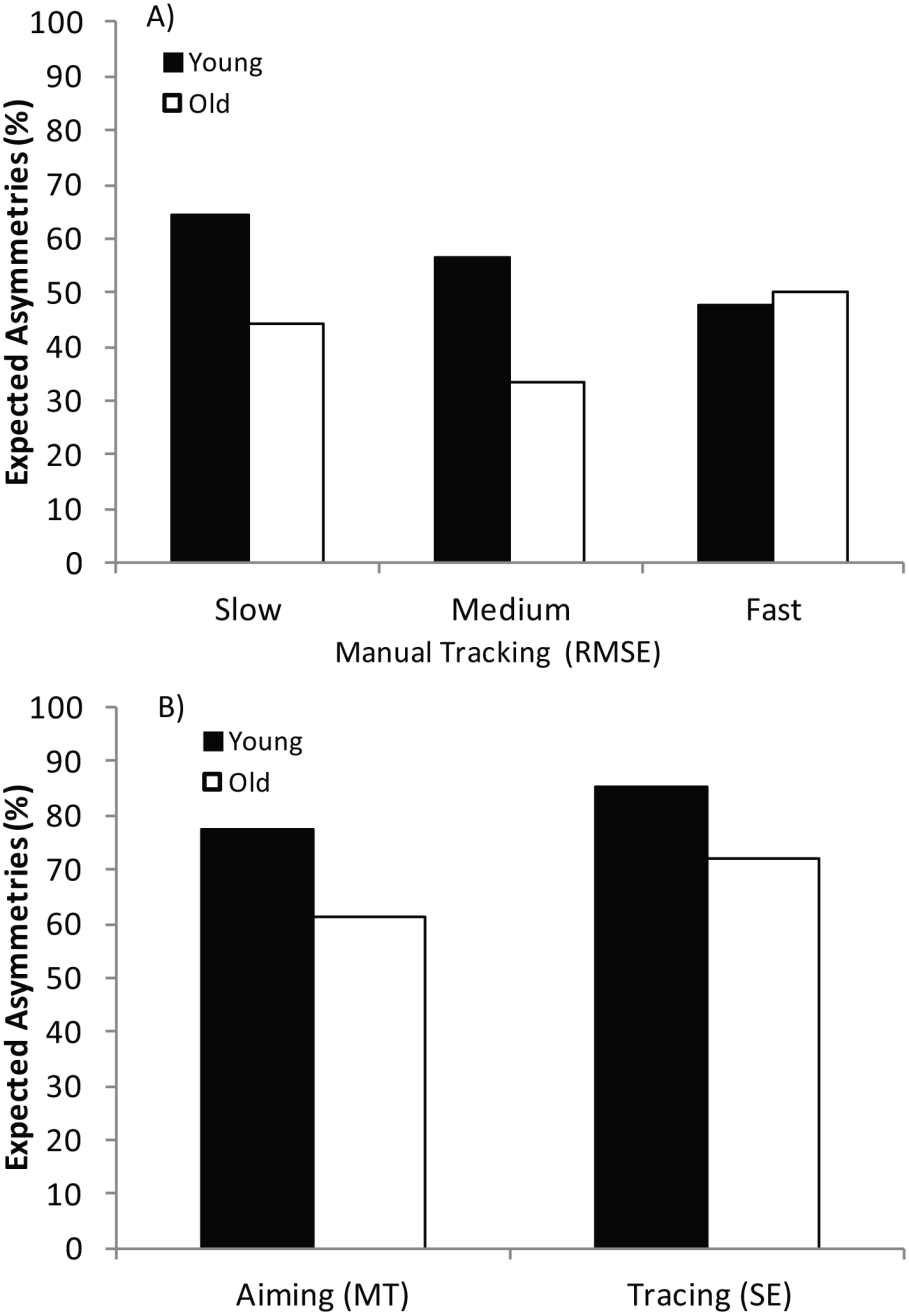
Manual Asymmetries in KineLab Motor Battery Tasks (Experiment Two). Proportion of young (black bars) and old (white bars) participants that showed manual asymmetries in the expected direction (i.e. preferred hand performing better) on measures of motor performance recorded during Experiment Two. For the Manual Tracking task, the combined RMSE values from both versions of the Manual Tracking Task (with and without spatial pattern) were averaged across the slow, medium and fast speed conditions. For Aiming and Tracing tasks the difference between the preferred and non-preferred hand were calculated for Movement Time (MT) or Shape Error (SE) respectively.

One possible limitation of the methods used to describe manual asymmetries so far is that they do not explain the degree to which individual differences influence the findings. Whilst the preferred hand was superior in some individuals (i.e. faster or more accurate) there were also cases where the preferred hand was actually worse. To examine the extent to which old and young participants conformed to expected asymmetries (i.e. better performance when using the preferred hand) we calculated an ‘asymmetry value’ between hands for each person in each task (i.e. performance for the preferred hand subtracted from the non-preferred hand). Fig 5 shows the proportion of young and old participants that produced the expected asymmetries (i.e. the % of participants with the preferred hand performing better) for the primary outcome measure recorded during the Manual Tracking, Aiming and Tracing tasks. For Manual Tracking, the combined RMSE values from both versions of the task (with and without spatial pattern) were averaged and then calculated for the slow, medium and fast speed conditions (Fig 5a). For the Aiming and Tracing tasks, the difference between the preferred and non-preferred hand was calculated for MT and SE, respectively (Fig 5b). Despite the fact that both age groups were classed as right-handed (i.e. mean EHI score for old = 99.44, *SD* = 2.36; mean EHI score for young = 97.26, *SD* = 8.28), not all participants demonstrated superior performance when using their ‘more-skilled’ hand. In the Manual Tracking Tasks even though there were no significant hand asymmetries revealed by the RMSE measure, approximately 50% of participants showed some degree of improvement using the preferred hand. Interestingly, the proportion of young participants displaying the expected hand asymmetries seemed to be related to the tracking speed: at slower tracking speeds a greater proportion exhibited the expected hand asymmetries (64%; *χ* ^*2*^ (1, *N* = 67) = 5.34, *p* = .02) whereas at fast speeds this was not the case (48%; *χ* ^*2*^ (1, *N* = 67) = 1.21, *p* = .27). This is consistent with increased task demands (at faster speeds) leading to poorer performance in the preferred hand and subsequent difficulties detecting manual asymmetries). While the majority of young did display some asymmetry in MT when Aiming (78%; *χ* ^*2*^ (1, *N* = 67) = 20.43, *p* <.001) this pattern was not evident in the old (61%; *χ* ^*2*^ (1, *N* = 18) = 0.89, *p* = .34). It should be noted there were still 22% of the young who did not show the expected MT asymmetries in the Aiming Task). Similarly while the majority of young did display some asymmetry in SE when Tracing (85%; *χ* ^*2*^ (1, *N* = 67) = 32.97, *p* <.001) this pattern was not as clear in the old (72%; *χ* ^*2*^ (1, *N* = 18) = 3.56, *p* = .06). Despite apparent asymmetries, 15% of the young and 28% of the old did not demonstrate the expected asymmetries in the Tracing Task. It seems then, that despite strong hand preferences (i.e.as indexed by EHI), there were large individual differences in the extent of manual asymmetries exhibited.

While all participants were indexed (by the EHI) as strongly right-handed, one plausible explanation for the individual differences found in manual asymmetries could be that those participants with a weaker preference for the right hand (i.e. lower EHI scores), were also those who showed smaller asymmetries. To examine this further we investigated whether there was a relationship between magnitude of asymmetries exhibited by participants and their degree of hand-preference. No significant correlations were found for RMSE in Manual Tracking (*r* (85) = -.100, *p* = .362), MT in Aiming (*r* (85) = .001, *p* = .994) or SE in Tracing (*r* (85) = -.148, *p* = .178). Note that there were also no correlations when the different age groups were examined separately, or when the different tracking speeds were examined separately. Furthermore, there were no correlations between chronological age of older adults and the degree of manual asymmetries.

### Discussion

In Experiment Two, age differences in motor performance were prevalent across all tasks. Older participants were less accurate when tracking, especially when demands were high (i.e. faster speeds); they took longer in the Aiming task, and showed a greater deviation from the ‘ideal’ reference path when Tracing. The critical question, however, was whether older adults would exhibit reduced manual asymmetries compared to the young. This was not the case, as very similar patterns of behaviour emerged for both young and old groups. In the Manual Tracking task neither group exhibited consistent hand asymmetries, whereas there were clear hand asymmetries in the Aiming and Tracing tasks. These data support the hypothesis that the relatively small differences between the preferred and non-preferred hand can be seen in some tasks but not others. Importantly, our tasks did not detect any age-related changes in manual asymmetries—unlike previous studies of this nature [15, 21, 36]. This opposes the theory that suggests changes in hemispheric lateralisation [12] can lead to reduced asymmetries at a behavioural level in older adults; as such a mechanism would predict reduced asymmetries regardless of task. Instead, these data support the hypothesis that differences between the preferred and non-preferred hand are relatively small (i.e. because mastering a skill involves learning the dynamical structure—a form of learning that benefits both hands), and asymmetries vary in magnitude as a consequence of task demands. Detecting these differences therefore requires (i) that tasks push both hands to perform at a high level of capability, and, (ii) that there is careful selection of the appropriate outcome metric. The latter is especially difficult if participants trade-off one aspect of performance (e.g. slower speed) for another (e.g. improved spatial accuracy). Experiment Three explores this issue of compensatory trade-offs in more detail.

## Experiment Three

Experiment One showed that the detection of manual asymmetries requires tasks that ensure participants are within the ‘Goldilocks Zone’; where it is possible to detect the relatively small performance differences between the two hands (which we suggest arise as a consequence of the lifelong processes of SL). A further issue with regard to the measurement of manual asymmetries was then highlighted in Experiment Two—when examining differences between the hands in special populations, such as older adults, it is important to explore a range of different outcome measures, particularly due to the possibility of motor strategies being applied in order to compensate for motor decline. Specifically, Experiment Two showed that in spite of the age-differences in overall performance (i.e. older adults showing decreased performance compared to young) there was no evidence of an age-related reduction in manual asymmetries (as reported in some previous studies) [15, 21, 36]. This highlights the importance of selecting the correct choice of outcome measure to index motor performance, as asymmetries might very well have been present in un-measured aspects of performance. For example, we have shown in the past that older adults often reduce speed in order to compensate for decreased spatial accuracy during tracing tasks [22]; therefore it is vital to consider both the temporal and spatial aspects of performance when examining differences between the hands in older people.

Experiment Three explored the issue of compensatory trade-offs in more detail with particular regard to the selection of the appropriate outcome metric. This experiment used a tracing task that was designed to capture manual asymmetries under different levels of temporal and spatial constraint. The tasks used in the test battery of Experiment Two (tracking, aiming and tracing) did not allow comparisons to be made between varied spatial and temporal constraints within the same task. For example, the aiming task allowed participants to trace at their own speed, but there was no spatial restriction on the route participants took when moving the pen between dots (i.e. they were just instructed to perform the task ‘quickly and accurately’). Furthermore, the tracing task imposed a temporal restriction, as participants had to keep their tracing within a moving ‘window’, independent of the spatial restriction (the thickness and shape of the path). To examine these issues in Experiment Three both the temporal and spatial components were explicitly and independently controlled.

### Methods

#### Participants

Another new opportunistic sample was recruited (from local community centres in Leeds), comprising twenty four right-handed individuals with a mean EHI score of 97.71 (*SD* = 4.82) and no self-reported history of ophthalmological or neurological problems. Eleven participants (8 female, 3 males) aged between 18 and 32 years formed the ‘young’ group (mean = 24.18, *SD* = 4.24) and 13 participants (9 female, 4 males) aged between 61 and 75 years formed the ‘old’ group (mean = 69.08, *SD* = 3.10).

#### Materials and Procedure

Three tracing tasks were created using KineLab [2], which required participants to draw along paths presented on a tablet PC using a handheld stylus (i.e. the same apparatus as used in Experiments One and Two). The paths featured were identical to those in Experiment One, each maintaining the same shape, but varying in thickness (2mm, 4mm, 6mm). In Experiment One, two temporal constraints were applied to create a ‘Slow’ and ‘Fast’ tracing version of the task. These two tasks were also included in this experiment, but with the addition of a ‘Preferred speed’ condition. The Preferred speed tracing task allowed participants to trace at their own pace (i.e. participants were instructed verbally to “trace at your preferred pace”), with no horizontal moving bars present to restrict them. In all conditions the path remained static and was fully visible throughout the trial. Each path thickness condition (i.e. narrow, medium and thick) was presented five times within each of the tasks (i.e. in the Fast, Slow and Preferred speed versions) resulting in a total of 45 paths to trace, presented in a random order. Instructions for participants appeared on the screen at the start of the task, and were similar to Experiment One, but with a reminder that participants must trace at their own pace when no bars appeared. All participants completed these task conditions once using their preferred (right) hand and once using their non-preferred (left) hand, which was counterbalanced so that every other participant began with their non-preferred hand. As in the first experiment, Shape Error (SE) was recorded as a measure of tracing accuracy (NB. higher scores = reduced accuracy), as well as a second metric, Movement Time (MT) as a measure of participants’ preferred movement speed in the unconstrained speed condition.

#### Analysis

For the analysis, the mean performance scores in the three path thickness conditions on each of the temporal versions of the task were calculated (i.e. SE for Fast and Slow speed tracing; SE and MT for Preferred speed tracing), and separate mixed ANOVAs were applied to examine differences between the task speed conditions, hands, and age groups. Missing data points were excluded (e.g. some spurious values were caused on occasion by participants’ accidentally touching the screen with their hand), but there were no more than two values excluded for each outcome measure of each participant. Greenhouse-Geisser estimates of sphericity (ε) are reported where degrees of freedom have been adjusted.

### Results

#### Fast Speed Tracing

There was a main effect of hand on SE (*F* (1, 21) = 9.35, *p* = .06, *η* ^*2*^_*p*_ = .31) whereby tracing was more accurate when using the preferred hand. There was also a reliable main effect of path thickness on SE (*F* (2, 42) = 8.47, *p* = .016, *η* ^*2*^_*p*_ = .29, *ε* = .74) with thicker paths producing worse compliance with the shape. This is consistent with previous findings of reduced corner-cutting with increased path thickness (22, 36). While age group differences approached significance (*F* (1, 21) = 4.11, *p* = .056, *η* ^*2*^_*p*_ = .16) there were no reliable interactions, which suggests that manual asymmetries were equivalent across both groups of participants and in all path thickness conditions.

#### Slow Speed Tracing

Patterns of SE were similar to those during Fast speed racing, with no reliable differences between the age groups. There were again significant effects of hand (*F* (1, 20) = 8.13, *p* = .01, *η* ^*2*^_*p*_ = .29) and path thickness (*F* (2, 40) = 83.08, *p* < .001, *η* ^*2*^_*p*_ = .81) whereby tracing performance was better when the path was narrow and when the preferred hand was used. It should be noted that this result differs slightly from Experiment One where the preferred hand was more accurate than the non-preferred hand in the Fast condition but not the Slow. This highlights again that caution is required when drawing conclusions from the absence or presence of hand differences. A lack of interactions once again demonstrates that manual asymmetries were equivalent across both age groups and all path thickness conditions.

#### Preferred Speed Tracing

**Fig 6:**
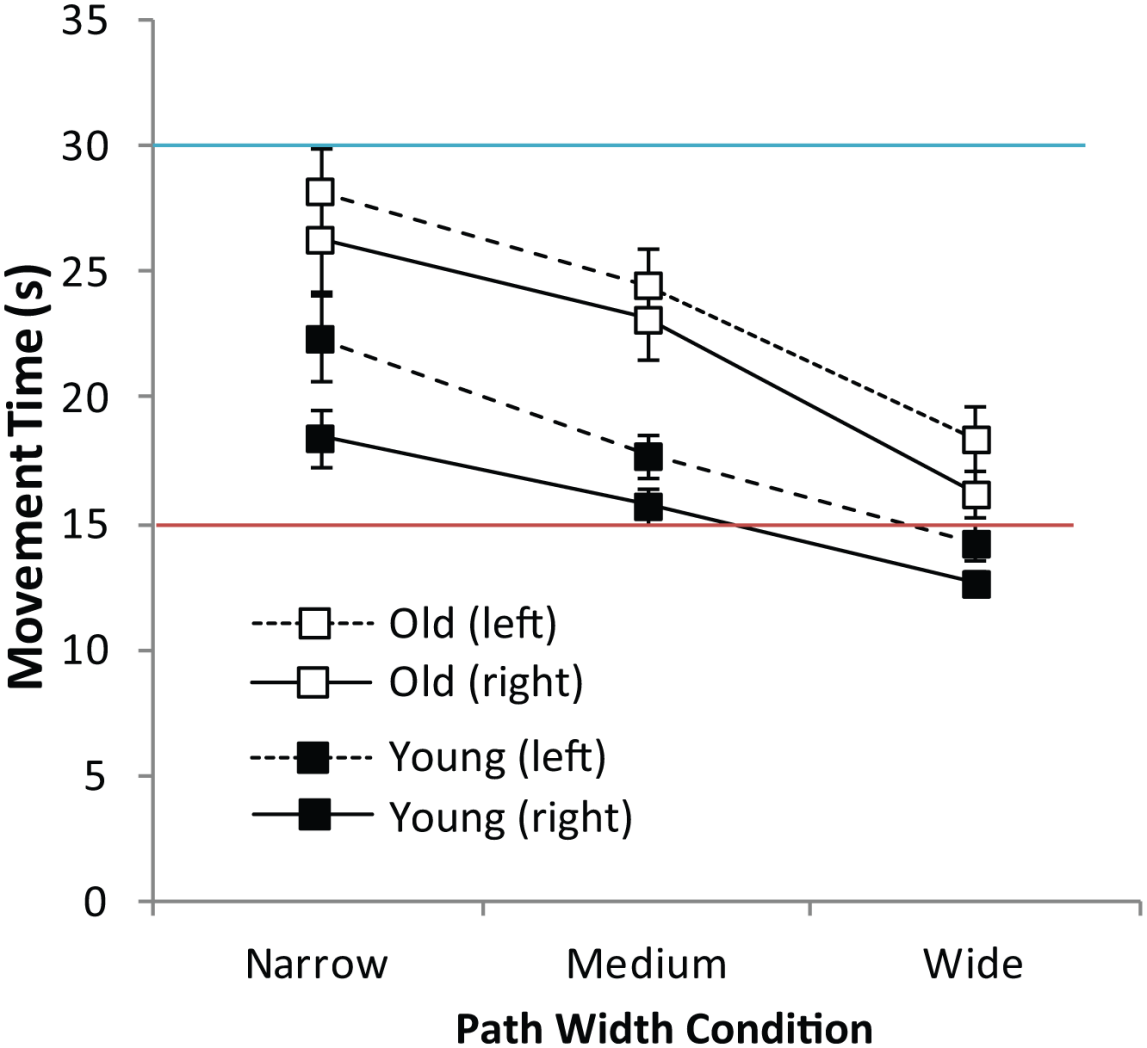
Movement Time in KineLab Tracing Task (Experiment Three). Mean Movement Time (MT) in seconds (s) for the narrow (2mm), medium (4mm) and thick (6mm) paths for the preferred hand (solid line) and non-preferred hand (dashed line), in the Preferred Speed Tracing Task in Experiment Three. The coloured lines indicate mean MT in the Slow Speed Tracing (blue) and Fast Speed Tracing (red) versions of the task. The bars represent the standard error of the mean (SEM).

Unlike the previous two tasks, Preferred speed tracing allowed participants to move at their own pace and hence employ speed-accuracy tradeoffs (i.e. increase MT to reduce SE). Consequently both SE and MT data were examined in turn. While increased path thickness impaired accuracy (i.e. increased SE; *F* (2, 40) = 196.04, *p* < .001, *η* ^*2*^_*p*_ = .91), there were no effects of age or hand, and no reliable interactions for the SE measure. Movement Times (MTs) on the other hand were affected by both age and hand condition. Fig 6 displays mean MTs for the young and old when tracing with the preferred and non-preferred hands on the narrow, medium and thick paths. Tracing was significantly faster on thicker paths (*F* (2, 42) = 75.38, *p* < .001, *η* ^*2*^_*p*_ = .78, *ε* = .69) and a reliable interaction between hand and path thickness (*F* (2, 42) = 3.73, *p* = .032, *η* ^*2*^_*p*_ = .115), revealed consistently slower tracing when the non-preferred hand was used, especially when the path was narrow. Older adults took significantly longer to trace paths compared to the young, evident in a significant main effect of age on MT (*F* (1, 21) = 13.75, *p* = .001, *η* ^*2*^_*p*_ = .40) nevertheless, an absence of any further interactions for the MT metric reinforces the suggestion that manual asymmetries in tracing speed were equivalent across both age groups in this version of the task.

### Discussion

The results from Experiment Three highlight an important issue when it comes to drawing conclusions about group differences based on performance of a task indexed by a single outcome measure. An obvious variable to use when examining tracing behaviour is Shape Error (SE), because it indicates the extent to which participants maintain the correct path throughout the trial. While SE did reveal hand differences, there were no age group differences observed. In contrast, when tracing speed was unconstrained (i.e. in the Preferred speed tracing task) a measure of Movement Time (MT) provided an additional metric that was able to reveal both hand and age-group differences.

If SE had been the only measure used to address the question of whether manual asymmetries exist, different tasks would have yielded opposing answers. There was evidence for manual asymmetries in SE on the Fast speed and Slow speed tracing Tasks (as well as the tracing component of the test battery in Experiment Two), yet SE did not reveal asymmetries when participants paced themselves in the Preferred speed version of the tracing task. The reason for this finding was made evident in the Movement Time (MT) data. Participants moved their non-preferred hand more slowly than their preferred hand, which allowed the two hands to perform at an equivalent level of accuracy. This speed/accuracy trade-off, also led to difficulties in detecting group differences, since the older participants slowed down their movements to a greater extent than the younger participants.

These results provide further evidence of participants strategically compensating for task demands. In all versions of the tracing task, the thicker the path being traced, the lower the participants’ SE score was. This finding confirms our previous reports of participants making spatial and temporal adjustments to their movements in order to meet task demands [22, 36]. The fact that different age groups make different compensatory adjustments [8, 22, 36] means that it is not simple to compare performance between these groups. Such measurement difficulties also make it harder to detect subtle performance differences between the hands.

## General Discussion

There is a conflict in the behavioural literature with regard to the nature and degree of manual asymmetries. Whilst from a young age humans have a strong phenomenological sense of being right or left-handed, mixed findings in previous research, particularly in studies of special populations (e.g. children with developmental disabilities), or with older adults with age-related motor decline, have led us to question the validity of this assumption. Studies that have compared manual asymmetries between old and young adults have revealed an inconsistent pattern of results. Some studies have shown no asymmetry differences between age groups [23–26] whereas others propose that manual asymmetries are absent or reduced in older adults [15, 21, 22]. Reduced asymmetries have been linked with neurophysiological observations of reduced hemispheric asymmetries in older adults [12, 14, 16–18]. The present series of experiments examined the degree to which the mixed pattern of results could be due to the subtle nature of manual asymmetries, which would be highly dependent upon task demands as well as the motor performance metric of choice.

In the first experiment, we empirically tested the concept of (what we label) a ‘Goldilocks Zone’; within which manual asymmetries are most likely to be detected. It was predicted that a motor task must have an appropriate level of complexity, being not too easy, nor too difficult, but just right. This zone became apparent in the Fast Speed Tracing Task, where the preferred hand performed with superior accuracy, but not when participants traced at a much slower pace in a different tracing condition. These findings demonstrate the ability of the non-preferred hand to match the accuracies of the preferred hand in a simple task, but with less proficiency under a strict temporal constraint. Note that we would expect a task with even greater spatiotemporal constraints to have led to reduced hand asymmetries; in that, a task that is too difficult for the preferred hand, would also fall outside of the ‘Goldilocks Zone’. A thought experiment shows that this prediction must be the case—if a task is too difficult to complete with either hand then it is obviously not possible to identify asymmetries.

Two further studies clearly indicated that reduced hemispheric asymmetries at the neurophysiological level does not account for the behavioural data: (i) evidence of similar manual asymmetries in younger and older adults was found; (ii) patterns of manual asymmetries for both younger and older adults were not constant across different tasks; and (iii) large individual differences in the measured manual asymmetries were found, despite participants reporting similarly strong hand preferences. These observations all support the notion that differences between the hands are relatively small and thus prone to difficulties of measurement. Furthermore, our findings are not consistent with the hypothesis that motor output is affected by changes in hemispheric specialisation. Generalisation of the hemispheric specialisation hypothesis [12] to motor cortex and motor output implies that reduced manual asymmetries should be reliably observed in older people across a range of motor tasks. The present study finds that older people show manual asymmetries in some specific tasks whilst other tasks led to no asymmetries in either younger or older adults.

Importantly, our work reconciles conflicting reports within the literature by demonstrating how empirical investigations of manual asymmetries are highly sensitive to task constraints (and individual differences within groups). There are a number of empirical studies that show reduced manual asymmetries in older adults [15, 21, 22] and these have been used to support generalisation of the hemispheric specialisation model to motor cortex and movement control. However, some research has failed to find any age differences in manual asymmetries [23–26], with others even reporting increased asymmetries in older adults compared to the young [24, 26–29]. It seems safe to conclude that the primary cause for differences in the existing literature simply relates to the nature of the constraints of the tasks and the outcome metrics used to explore the magnitude of differences in hand performance. There are a variety of factors that could make a task more difficult (e.g. introducing novel force fields) but novel tasks often differentially impair preferred hand performance because the manipulations can disrupt the finely honed visual-motor relationships employed by the preferred hand. Tasks that reveal improved performance in the preferred hand will, therefore, have elements of well-learned behaviours within them, whilst also including some spatio-temporal constraints to ensure they are not too easy for the non-preferred hand. The best example across our experiments is the tracing task, which consistently revealed hand asymmetries (except during enforced slow conditions in Experiment One), which is sufficiently difficult to demonstrate the superior performance of the preferred hand.

The fact that different tasks yield different patterns of asymmetry highlights two important issues with regards to the way in which manual asymmetries are examined. First, the process of capturing hand differences requires a task that yields optimal performance with both hands (i.e. a task within the ‘Goldilocks Zone). Secondly, previous studies of age differences in manual asymmetries have often used combined speed-accuracy measures, or relied on speed as the only marker of performance. This is problematic because spatial and temporal compensatory adjustments can then be missed. It is therefore essential not to base conclusions about group manual asymmetry differences on just one outcome metric—one metric may miss effects that are manifest in other (unmeasured) aspects of performance. The problem of potentially missing effects present in unmeasured aspects of performance is particularly germane when studying the movements of older people. The tasks that were used with younger and older groups in Experiment Three varied both the temporal and spatial constraints. In the Slow and Fast speed tracing tasks the aim was to maintain spatial accuracy at the required speed. Shape Error (SE) was therefore selected as the marker of performance because it captured the extent to which participants maintained the shape of the path as they traced. Subsequent analyses revealed that participants were more accurate when tracing with the preferred hand, but with only marginal age differences (between-subjects effect, *p* = .056). Given the significant age differences observed in Experiment Two it seems plausible that a larger sample size would have led to significant age differences. Furthermore, when participants were free to move at their Preferred speed, age differences were revealed, but only in the MT metric (not in the measure of SE). It seems that older participants preferred to maintain their accuracy and trace at a slower pace, especially when using their non-preferred hand. We would suggest that the older adult group were able to effectively compensate and match the accuracy of the young by slowing movements down, which confirms previous findings that participants can make strategic compensatory spatial adjustments to account for task demands [22, 36].

The argument that manual asymmetries are subtle and difficult to measure does not in any way suggest that manual asymmetries do not exist. The Edinburgh Handedness Inventory (EHI) clearly indicated a strong hand preference across the vast majority of our participants. Moreover, participants frequently reported how much more difficult they found the task when using their non-preferred hand. Nevertheless, all participants were capable of completing the tasks with their non-preferred hand despite limited experience of holding a stylus (e.g. pen) with this hand. So what might explain this contradiction between what we ‘sense’ as our preferred hand, and our surprising ability to perform well in some conditions when using our ‘weaker’ hand? We would attribute this phenomenon to the consequences of Structural Learning (SL). This framework suggests that the control dynamics of holding a stylus and generating the appropriate forces are learned at an abstract effector-independent level. These control dynamics can then be exploited when generalising the skill—in this case to the non-preferred hand. The dynamics of controlling a stylus in the non-preferred hand will not be identical to those involved in the preferred hand but there will clearly be large similarities. The ability to generalise control dynamics will not allow the highest level of performance to be achieved (it seems reasonable to assume that would require direct trial-and-error learning of the precise dynamics of the particular limb), but would ensure a reasonable level of performance. It follows that learning with one hand will be transferred to the other hand and thus necessitate sensitive measures to detect performance differences. Our present findings support this view. It seems that our lifelong tendency to use our preferred hand to complete fine motor control tasks allows us to develop skills that can be easily applied to the non-preferred hand, to a similar level of proficiency, depending on task demands.

Research into the mechanisms of SL suggests that the extent to which the non-preferred hand is able to match the proficiency of the preferred hand, could actually be mediated by the nature of the control mechanisms engaged by the task. Yousif and Diedrichsen [34] argued that SL varies depending on whether tasks were feed-forward (i.e. predictive) or feedback (i.e. corrective) based. Their study explored whether a single mechanism underpinned adaptation to the feed-forward and feedback component of movements made within a perturbed force field, and found that the structural responses to force perturbations were at least partly dissociable for feed-forward and feedback control. The work by Yousif and Diedrichsen [34] implies that the nature of SL will vary between tasks as a function of the extent to which a task is based on feed-forward or feedback control. With regard to the issue of inter-manual transfer, this finding reinforces the idea that disparate tasks will yield different manual asymmetries.

In conclusion, our results provide an alternative explanation for different patterns of manual asymmetries observed across various experimental tasks. Manual asymmetries can be missed if the task does not place participants within, what we have named, the ‘Goldilocks zone’. These data suggest that caution must be exercised before conclusions are drawn from findings of reduced manual asymmetries. A number of authors have proposed (in a similar way to Orton [30]) that reduced manual asymmetries indicate an ‘underlying confusion of cerebral dominance’. We suggest that such conclusions regarding brain function are not merited on the basis of reduced asymmetries recorded within an experimental task unless it can be demonstrated unequivocally that the task placed participants within the ‘Goldilocks Zone’.

The tasks used across all three experiments in the present work have allowed us to (i) demonstrate the presence of a ‘Goldilocks Zone’ predicted to exist within the context of SL; (ii) provide evidence of task-dependent manual asymmetries in older adults, which is a controversial finding within the ageing literature; (iii) establish that different tasks yield different patterns of asymmetries in both younger and older adults; and (iv) identify individual differences in the measured manual asymmetries despite participants reporting similarly strong hand preferences. These observations support the notion that differences between the hands are relatively small and hence prone to difficulties of measurement as predicted by the lifelong processes of SL.

The fact that different tasks yield different patterns of asymmetries highlights two important issues with regards to the way in which manual asymmetries are examined. First, the process of capturing hand differences requires a task that yields optimal performance with both hands. Second, previous studies of age differences in manual asymmetries have often used combined speed-accuracy measures, or relied on speed as the only marker of performance. This is problematic because spatial and temporal compensatory adjustments can then be missed. It is essential not to base conclusions about group manual asymmetry differences on one outcome metric—one metric may miss effects that are manifest in other (unmeasured) aspects of performance. The problem of missing effects in unmeasured aspects of performance is particularly germane when studying movement in special populations (e.g. older adults).

## Supporting Information

S1 Dataset Data for all experiments. (XLSX)
